# Enhanced adsorptive removal of p-nitrophenol from water by aluminum metal–organic framework/reduced graphene oxide composite

**DOI:** 10.1038/srep25638

**Published:** 2016-05-16

**Authors:** Zhibin Wu, Xingzhong Yuan, Hua Zhong, Hou Wang, Guangming Zeng, Xiaohong Chen, Hui Wang, Lei zhang, Jianguang Shao

**Affiliations:** 1 College of Environmental Science and Engineering, Hunan University, Changsha 410082, P. R. China; 2 Key Laboratory of Environment Biology and Pollution Control, Hunan University, Ministry of Education, Changsha 410082, P. R. China; 3 Department of Soil, Water and Environmental Science, the University of Arizona, Tucson, AZ85719, US; 4 Hunan University of Commerce, Changsha 410205, P. R. China

## Abstract

In this study, the composite of aluminum metal–organic framework MIL-68(Al) and reduced graphene oxide (MA/RG) was synthesized via a one–step solvothermal method, and their performances for p–nitrophenol (PNP) adsorption from aqueous solution were systematically investigated. The introduction of reduced graphene oxide (RG) into MIL-68(Al) (MA) significantly changes the morphologies of the MA and increases the surface area. The MA/RG-15% prepared at RG-to-MA mass ratio of 15% shows a PNP uptake rate 64% and 123% higher than MIL-68(Al) and reduced graphene oxide (RG), respectively. The hydrogen bond and π – π dispersion were considered to be the major driving force for the spontaneous and endothermic adsorption process for PNP removal. The adsorption kinetics, which was controlled by film–diffusion and intra–particle diffusion, was greatly influenced by solution pH, ionic strength, temperature and initial PNP concentration. The adsorption kinetics and isotherms can be well delineated using pseudo–second–order and Langmuir equations, respectively. The presence of phenol or isomeric nitrophenols in the solution had minimal influence on PNP adsorption by reusable MA/RG composite.

Nitrophenols are widely used in petrochemical synthesis, including paints, plastics, rubber, pulp, pesticides and dyes production^1^. The presence of nitrophenols in the industrial wastewater has aroused great concerns in recent years due to the increase in wastewater discharge and the toxicity of nitrophenols to the receiving bodies^2^. In particular, the p-nitrophenol (PNP) has intensive toxic effect on methaemoglobin formation, causing liver and kidney damage, anaemia, skin and eye irritation, and systemic poisoning^3^,^4^. It has been listed as a priority pollutant by the U. S. Environmental Protection Agency (U.S. EPA)^5^. For years, to minimize nitrophenol pollution from wastewater, the methods of photo-degradation^6^, adsorption^7^, and chemical oxidation^8^, etc, have been developed. Among these methods, adsorption is considered to be a promising one due to the advantages of this method, e.g., simplicity and cost-effectiveness.

Graphene oxide (GO), a type of negatively charged colloid comprising multiple oxygenated graphene layers with one-atom thickness honeycomb lattice structure, has received great attention for pollutants removal from wastewater due to the high specific surface area and great application promise^9^,^10^,^11^,^12^. For example, *Wan*g *et al.*^13^ used reduced graphene oxide for adsorption of phenolics and interpreted the correlation between the adsorption ability and reduction degree of graphene oxide. In our previous studies, the graphene oxide exhibited excellent efficiency for Zn^2+^ removal^14^ and a superior adsorption capacity of methylene blue was achieved by rhamnolipid functionalized graphene oxide^15^. In terms of the removal of PNP, *Zhang et al.*^16^ reported that the precursor for GO, nanographite oxide, has a maximum PNP adsorption capacity of 268.5 mg/g at 283 K and a natural pH. However, the GO is hard to separate from solution after adsorption due to the hydrophilic property. Recently, reducing the surface functional groups of graphene oxide is considered to be an effective method to decrease hydrophilicity and thus achieve better separation performance^17^. Unfortunately, when the GO was reduced, the highest adsorption capacity of PNP was observed to be only 15.5 mg/gat 298 K and pH6^18^. Therefore, it is a challenge to improve the adsorption performance of graphene-based materials for PNP removal.

Due to the large surface area, diverse structure, and tunable functionality, metal–organic frameworks (MOFs) have recently attracted extensive attentions in adsorption, catalysis, sensing, gas storage, and drug delivery^19^,^20^,^21^. The MOFs of MIL–68(Al) (MA) is assembled from the infinite straight chains of corner–sharing metal–centered octahedral AlO_4_(OH)_2_ that is connected to each other through hydroxyl groups and terephthalate ligand^22^. *Yang et al.*^23^ reported that the MIL–68(Al) has great gas adsorption due to the presence of triangular and hexagonal channels of an opening diameter (6.0 ~ 6.4 Å and 16 ~ 17 Å). *Xie et al.* utilized MIL–68(Al) for nitrobenzene removal from water and achieved a quite high adsorption capacity of 1130 ± 10 mg/g^24^. The adsorption properties of MIL-68(Al) can be further improved by hybridation with other materials. *Han et al.*^25^ chose the functionalized carbon nanotube (CNT) to composite with MIL-68(Al) and exhibited 188.7% enhanced phenol adsorption capacity from water than pristine MIL-68(Al). Although series of MOFs/graphene based composites were synthesized and used for gas adsorption^26^, gas storage^27^, and organic compounds adsorption^28^,^29^, to date the composite of reduced graphene oxide (RG) and aluminum based MOFs and its application in pollutant removal from wastewater have not been reported.

In this study, the MIL–68(Al)/RG composite was synthesized using a simple solvothermal method, and the its performance for adsorptive removal of PNP from water was examined. The adsorption kinetics and thermodynamics were investigated in detail. Factors that may potentially affect the adsorption process, such as pH, ionic strength, temperature, recycle number and coexistence of isomers or phenol, were also examined.

## Result and Discussion

### Characterizations

**Figure 1:**
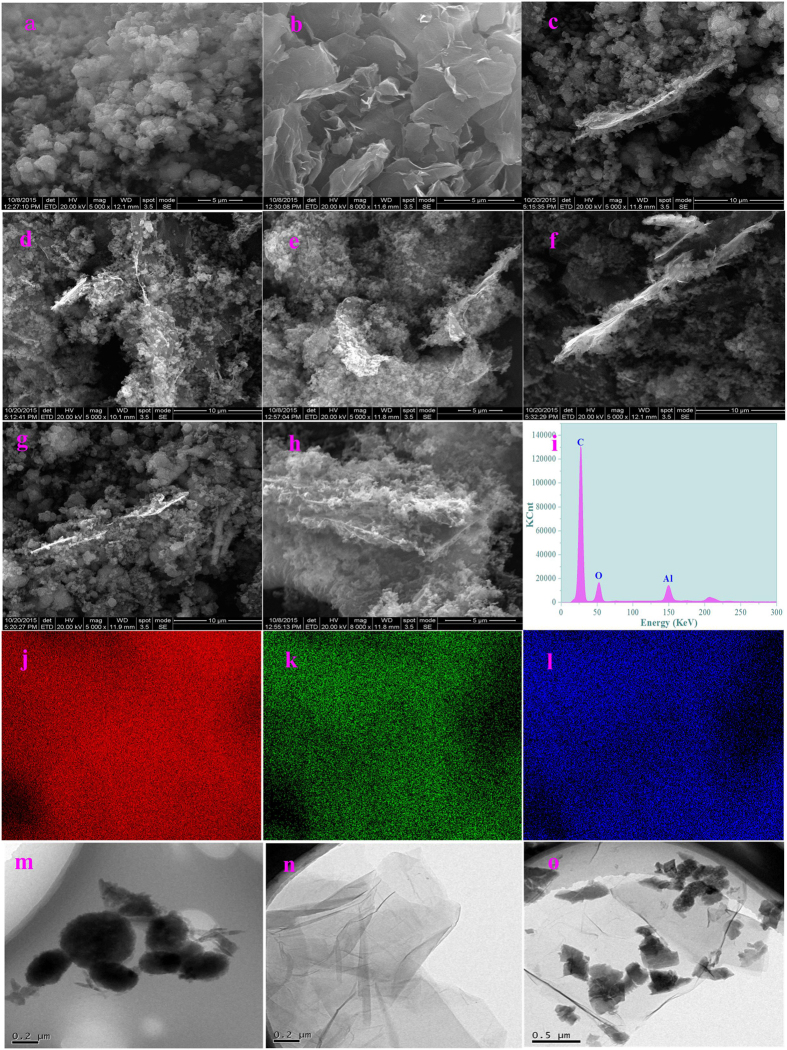
The SEM images of MA (**a**), RG (**b**), MA/RG–1% (**c**), MA/RG–5% (**d**), MA/RG–15% (**e**,**h**), MA/RG–25% (**f**), MA/RG–35% (**g**); The EDX spectrum (**i**) and elemental mapping images (**j**–**l**) of MA/RG–15%: C (**j**), O (**k**) and Al (**l**); The TEM images of MA (**m**), RG (**n**) and MA/RG–15% (**o**).

The morphologies of MA, RG and MA/RG observed by scanning electron microscopy (SEM) and transmission electron microscopy (TEM) are presented in Fig. 1. It can be seen that the MA (Fig. 1a) is in aggregative ball–liked particles and the RG (Fig. 1b) shows a flake–liked structure with wrinkles. After the RG composited with MA (Fig. 1c–h), the MA particles are observed to attach to the surface of RG layers, and the particle size appears to be smaller. During the preparation of MA/RG, the oxygen–containing groups including –COOH and –OH on the frame of GO were sufficient coordination with the Al^3+^, which provided the dense and homogeneous nucleation nods for the formation of MIL–68(Al) with precious few agglomeration, and thus leading to the formation of nanosized and well–dispersed MIL–68(Al) crystallites^19^,^30^. The results of element composition (Fig. 1i) and the maps of elements (Fig. 1j–l) obtained using EDX show that the C, O and Al are uniformly distributed on the surface of the MA/RG–15% (Fig. 1h). The TEM images (Fig. 1(m–o)) further confirm the morphological change after the hybridization. As depicted in Fig. 1o, the MA scattered in the transparent folded RG layers of MA/RG–15% composite are plate–like particles, which seemed to be different from the parental one (Fig. 1m), attributing to the distortion force of GO for MIL–68(Al) formation by the π–π stacking interaction^31^.

**Figure 2:**
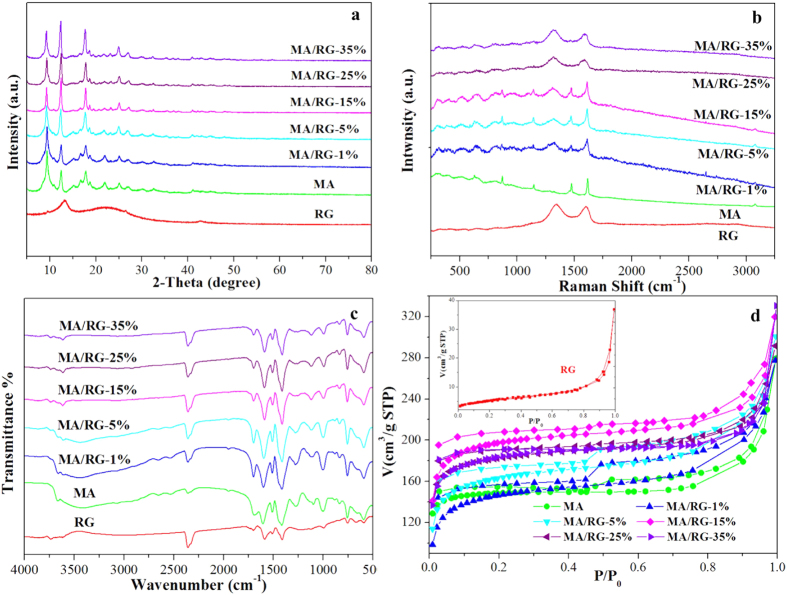
The XRD spectrum (**a**), Raman spectrum (**b**), FT–IR spectrum (**c**), and N_2_ adsorption – desorption isotherms (**d**) of MA/RG composites.

Figure 2a shows the X–ray diffraction (XRD) patterns of MA, RG and MA/RG composites. The characteristic peaks of MA are in agreement with the previous report of MIL–68(Al)^25^, suggesting that the current material has the structure as expected. For RG, the peak at 2θ = 22.2° corresponds to graphene with the interlayer distance of 0.400 nm, indicating the reduction of graphene oxide and the restoration of sp^2^ bonded carbon. The peak at 2θ = 13.2° is produced by the residual oxygen groups such as epoxy as carbonyl groups, due to incomplete reduction. After hybridization of MA with RG, the major diffraction patterns of the composites are similar to that of the pure MA, and no diffraction peak for RG is observed in the composites, which is probably due to that the low content of RG is shielded by the attached MA particles^32^. To testify the presence of RG, the Raman spectra (Fig. 2b) of the MA/RG composites are measured. The major characteristic peaks for RG include D peak (∼1348 cm^−1^) which is resulted from the breathing mode of κ–point phonons of A1 g symmetry, and the G peak (∼1601 cm^−1^) which is from the E_2g_ phonon of the sp^2^ hybridization^27^. For MA, the characteristic peaks at 1616, 1475 and 1147 cm^−1^ correspond to the in–plane vibration of the aromatic rings in the terephthalic acid ligands, and the peaks at 869 and 631 cm^−1^ are associated with C–H stretching or out–of–plane vibration of the aromatic rings^33^. Those peaks of MA and the D band of RG are also observed in the MA/RG composites, indicating successful hybridization of MA and RG. The MA/RG (MA/RG–1% to MA/RG–15%) present a shoulder band between 1594 and 1608 cm^−1^ and eventually show G peak of RG with the increase amount of RG (MA/RG–25% and MA/RG–35%), which is attributed to the integration of G band of RG with 1616 cm^−1^ peak of MA and the larger number of sp^2^ composite aromatic rings in its structure with more incorporated RG.

The FT–IR results for the parent materials and MA/RG composites are presented in Fig. 2c. Characteristic peaks of C–H (754 cm^−1^), C–O–C (991 cm^−1^), C=O (1512 and 1701 cm^−1^) and C=C (1411 and 1587 cm^−1^) are observed for RG^34^. These peaks are also observed in the spectra of MA and MA/RG composites. Different from RG, the MA and MA/RG has the other peaks of C–OH for carboxyl (1274 cm^−1^), C–H for benzene ring (1097 cm^−1^) in terephthalic acid ligand and –OH (3425 cm^–1^)^35^. The N_2_ adsorption–desorption isotherms obtained at 77 K are shown in Fig. 2d. All curves are type II isotherms typically with a type H3 hysteresis, due to the presence of mesopores^36^. It should be noted that the N_2_ ad-desorption isotherms for MA/RG were above that of the MA and RG, indicating that the specific surface area of the material is increased after MA composited with RG. The MA/RG–15% has the highest specific surface area. The porous structure parameters and the pore size distributions were obtained from the N_2_ adsorption data analyized using Barrete–Joynere–Halenda (BJH) model, and the results are summarized in Table 1. The MA/RG–15% exhibits the largest volume of micro and mesporous, which is consistent with the result of BET surface area measurement.

**Figure 3:**
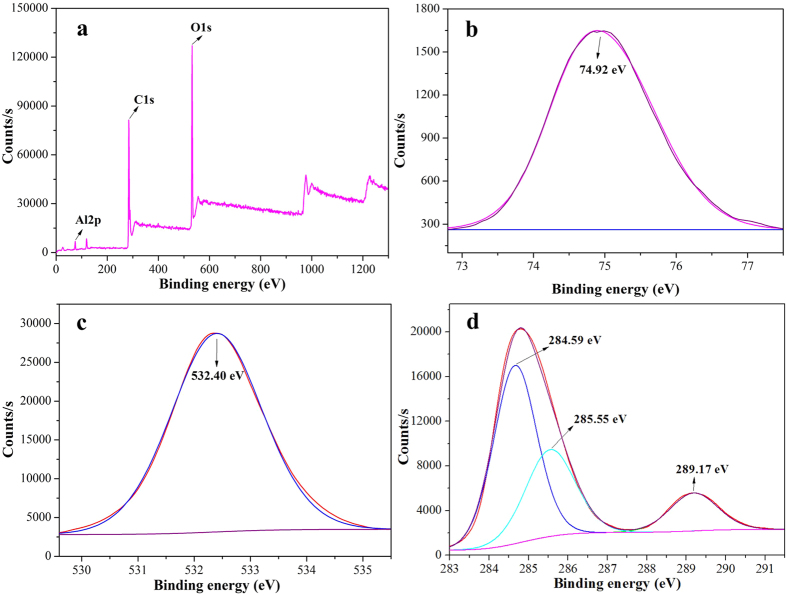
The XPS spectra and of MA/RG–15%: (**a**) the full XPS spectra; the core level spectra of Al2p (**b**), O1s (**c**) and C1s (**d**).

The surface element of MA/RG–15% is analyzed using the X–Ray photoelectron spectroscopy (XPS) and the results were presented in Fig. 3a. The atomic content of Al, C and O is 6.35%, 63.30% and 30.35%, respectively. The binding energy of the metal A12p is 74.92 eV (Fig. 3b), which is due to the formation of AlO_4_(OH_2_) in the MIL–68(Al) framework. The O1s peak (Fig. 3c) at 532.40 eV accounts for the carboxylate oxygen −COO of terephthalic acid and the residue oxygen–containing groups in RG in the composites. This is further confirmed by the C1s band (Fig. 3d), which can be divided into three peaks located at 284.59, 285.55, and 289.70 eV, corresponding to the C=C/C−C, C−O and carboxylate carbon structures, respectively^37^.

### The p**–**nitrophenol (PNP) adsorption

#### Adsorption kinetics and rate–control mechanism

**Figure 4:**
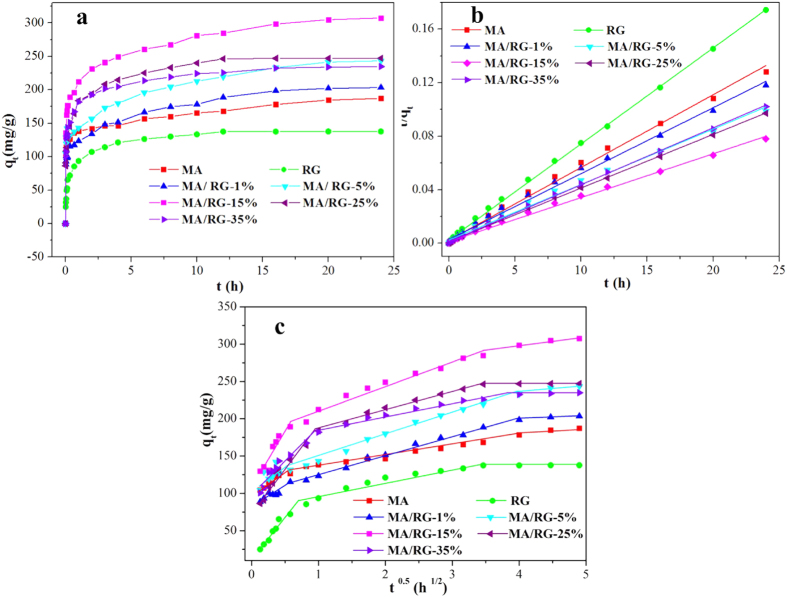
(**a**) The effect of time on PNP adsorption; (**b**) Pseudo–second–order plots for PNP adsorption; (**c**) Intra–particle diffusion for PNP adsorption.

The effects of contact time on the PNP adsorption on the RG, MA and MA/RG composites are shown in Fig. 4a. For all samples, the PNP adsorption rates decrease with time until the adsorption equilibrium is reached. After hybridization of MA with RG, the adsorption of PNP are significantly enhanced, and the MA/RG–15% exhibits the best adsorption performance of 307.38 mg/g, which is 64% and 123% higher than that of MA and RG, respectively, due to the increase of surface area. The molecular size of PNP is calculated to be 0.66 nm × 0.43 nm^38^, which is smaller than the average diameter of the pores (Table 1) of MA/RG composites. Therefore, the PNP molecules can easily enter into the pore and access the surface, which favors the PNP adsorption^39^.

The Pseudo–first–order^16^, Pseudo–second–order^40^ and Elovich^41^ equations are used to describe the adsorption kinetics (Eqs 1–3).


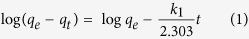



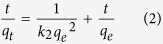



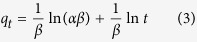


where *q*_*e*_ and *q*_*t*_ (*mg*/*g*) are the adsorption amount of PNP at equilibrium and time *t*(*h*), respectively. *k*_*1*_ (*h*^–1^) and *k*_*2*_ (*g*/(*g.h*)) are the Pseudo–firs–order and Pseudo–second–order adsorption rate constants, respectively. *α* (*mg*/(*g.h*)) is the initial sorption rate and *β* (*g*/*mg*) is related to the extent of surface coverage and activation energy for chemisorptions. Table 2 summarizes the adsorption kinetic parameters of PNP onto the tested adsorbents. Comparing with the correlation coefficients (*R*^*2*^) of the Pseudo–first–order, Pseudo–second–order and the Elovich models, it can be concluded that the Pseudo–second–order kinetic model fits the adsorption process of all samples better than the other two. Furthermore, the deviation between calculated *q*_*e,cal*_ and experimental *q*_*e,exp*_ values of the Pseudo–second–order kinetic model are very lower, while that of the Pseudo–first–order kinetic model is very large. The fitting line of Pseudo–second–order is perfectly plotted in Fig. 4b, suggesting that the adsorption–determining factor of the PNP removal may be involve in the chemisorption.

To better understand the diffusion rate controlling procedure, the Intra–particle diffusion model^42^ is tested as Eq. (4).


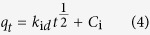


In which, *i* is the number of linear piecewise, *k*_*id*_ is the Intra–particle diffusion rate constant (*mg*/(*g.h*^*1*/*2*^)), and *C*_*i*_ is the intercept related to the thickness of the boundary layer. If the data of the whole adsorption process is good linear fit to intra–particle diffusion (i is only equal to 1) and *C* is zero, the intra–particle diffusion is the lonely rate limiting step, otherwise, the larger the intercept, the greater the contribution of the film diffusion sorption in the rate controlling^43^. As shown in Fig. 4c, the entire PNP adsorption process shows three linear sections in a curve, suggesting multiple steps take place during adsorption process.The piecewise fitting parameters of the Intra–particle diffusion are listed in Table 2. The values of *C*_*i*_ for each linear portion are not zero and the correlation coefficients (*R*_*2*_)^2^ show the highest value among than (*R*_*1*_)^2^ and (*R*_*3*_)^2^, indicating that intra-particle diffusion participate in the PNP adsorption controlling, but is not the sole rate-controlling step in all the stages, the film diffusion may also involve in the adsorption process. At the beginning of adsorption (the first segment in Fig. 4c), the film diffusion charged the mass transfer of PNP from the bulk solution to the external surface of MA/RG. As the adsorption processing (the second stage of Fig. 4c), adsorption rate starts to slow down and the intra–particle diffusion conducts the diffusion of the PNP molecules from the external surface into the pores of the MA/RG, which is also accompanied by film diffusion. In the end (the small slope section in Fig. 4c), the adsorption is reached equilibrium and the intra–particle diffusion fades out the PNP adsorption.

#### Adsorption isotherms and thermodynamics

**Figure 5:**
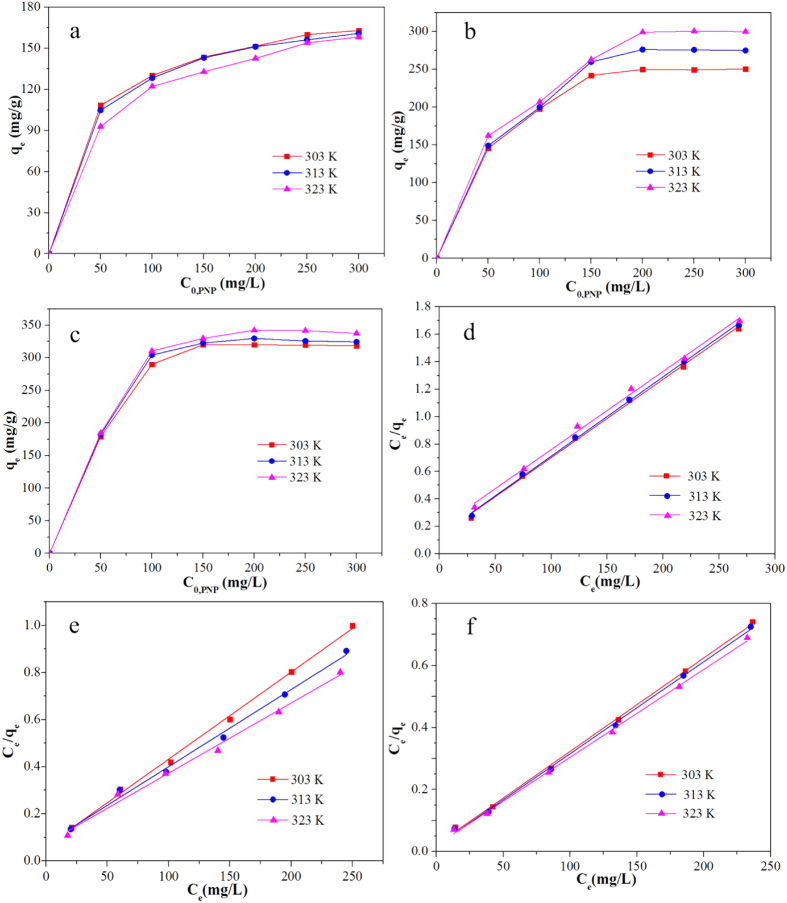
The PNP adsorption by RG (**a**), MA (**b**) and MA/RG–15% (**c**) at different temperature; The Langmuir isotherm model for PNP adsorption by RG (**d**), MA (**e**) and MA/RG–15% (**f**).

The adsorption isotherm investigations are carried out under different temperature with various initial PNP concentrations. As illustrated in Fig. 5(a–c), the uptake amount of PNP increases firstly with the increase of PNP concentration and then it keeps on a horizontal (the adsorption saturation stage). The reason may be that the higher initial PNP concentration, the more strength driving force provides to overcome the mass transfer resistances when the utilization of active sites do not reach adsorption saturation. Besides, for MA (Fig. 5b) and MA/RG–15% (Fig. 5c), the PNP adsorption amount increases with increase of temperature, while for RG (Fig. 5a), the adsorbed PNP is slightly decrease as increase in temperature, which suggests that an endothermic process is for MA and MA/RG–15%, but an exothermic procedure is for RG in nature. Therefore, after MA incorporated with RG, rising the temperature favors the PNP adsorption^44^.

To gain the insight into adsorption thermodynamic behavior, the Langmuir^45^ and Freundlich^18^ isotherm models are used to analyze the equilibrium data according to the Eqs (5) and (6), respectively.


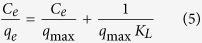



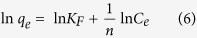


where, *C*_*e*_ is the equilibrium concentration (mg/L) of the PNP, *q*_*e*_ is the amount (mg/g) of the PNP adsorbed at equilibrium and *q*_*max*_ is the maximum adsorption capacity (mg/g), *K*_*L*_ (L/mg) is the Langmuir constants related to energy of the adsorption. *K*_*F*_ (L/mg) and *1*/*n* are Freundlich constants giving an indicator of the adsorption capacity and the adsorption intensity, respectively. Table 3 lists isotherm parameters of Langmuir and Freundlich isotherms for the PNP adsorption. It can be found that, for MA, RG or MA/RG, the regression coefficients *R*^*2*^ obtained from Langmuir model are much higher than that from Freundlich isotherm model, which suggests that the adsorption of PNP is best fitted with the Langmuir isotherm and the adsorption behavior is governed by monolayer adsorption on a homogenous surface. The linear relation between *C*_*e*_/*q*_*e*_ and *C*_*e*_ of Langmuir model is well ploted in Fig. 5(d–f). The maximum uptake capacity (*q*_*m,cal*_) calculated from Langmuir model of the MA/RG composite at different temperature are much higher than that of the parent MA and RG. The comparisons of PNP adsorption maximum capacity with various adsorbents previously reported are listed in Table 4. It can be seen that the MA/RG composite exhibits superior PNP uptake capacity than NiAl-layered double hydroxide^1^, alumina hollow microspheres^46^, NH_2_-MIL-101(Al)^47^, carbon nanotube^3^, nanographite oxide^16^ and graphene^18^, indicating that the MA/RG has great potentials for PNP removal from contaminated water.

To gain insight into the essential feature of Langmuir isotherm, a dimensionless constant separation factor (*R*_*L*_) is tested as the following equation 1:


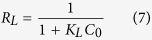


where, *K*_*L*_ (L/mg) is the Langmuir constant and *C*_*0*_ (mg/L) is the initial concentration of PNP in the liquid phase. The value of *R*_*L*_ indicates whether the type of the Langmuir isotherm is unfavorable (*R*_*L*_ > 1), linear (*R*_*L*_ = 1), favorable (0 < *R*_*L*_ < 1) or irreversible (*R*_*L*_ = 0). From Table 3, the *R*_*L*_ values for MA, RG and MA/RG are between 0 and 1, especially for MA/RG, the *R*_*L*_ values are smaller than that for the monomers, indicating that a more favorable adsorption process is for the PNP adsorption onto the MA/RG composites.

The in–depth information on inherent energetic changes related to the adsorption is provided by thermodynamic analysis. The Gibbs free energy change (*∆G*^*0*^) of the adsorption can be calculated by the following equation^48^:


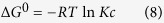


where, *R* is the gas constant (8.314 J/mol.K), *Kc* is distribution coefficient (*Kc* = *q*_*e*_/*C*_*e*_) at the specific temperature (K). As given in Table 5, the values of *∆G*^*0*^ are found to be negative at the tested temperature, indicating that the uptake process of PNP by RG, MA and MA/RG is spontaneous nature. By assuming that ***∆**H*^*0*^ and entropy change (*∆S*^*0*^) are independent of temperature for the adsorption processes with temperatures not significantly change, the values of ***∆**H*^*0*^ and *∆S*^*0*^ can be evaluated by linear regression of *∆G*^*0*^ versus *T* as Gibbs–Helmholtz equation^49^:


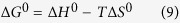


As presented in Table 5, the value of ***∆**H*^*0*^ is positive for MA and MA/RG composite, and negative for RG, which indicates that the PNP adsorption on MA and MA/RG is an endothermic nature, but on the RG is an exothermic process. This coincides with the result of temperature effects. Furthermore, the positive (or negative) value of *∆S*^*0*^ reflects an increase (or decrease) in randomness at the solid/solution interface for MA and MA/RG (or RG) during the PNP adsorption process^50^.

#### Effects of pH and ionic strength

**Figure 6:**
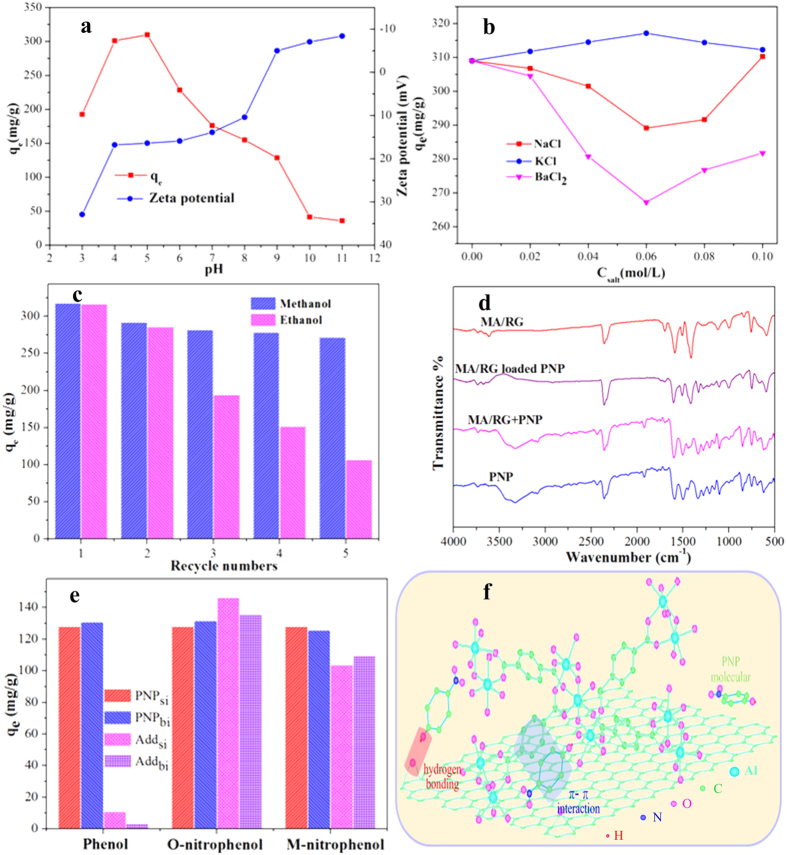
(**a**) the initial solution pH and (**b**) the ionic strength in the solution; (**c**) Reusability of the MA/RG for PNP adsorption; (**d**) The FT–IR spectra of MA/RG, PNP, MA/RG + PNP (mixture of MA/RG and PNP solid) and MA/RG adsorbed PNP; (**e**) The additives (phenol, ONP and MNP) adsorption in single (Add_si_) or binary (Add_bi_) of PNP and influence on PNP adsorption in binary (PNP_bi_) compared with in single solution (PNPsi); (**f**) The conceptual diagram of adsorption mechanism.

The effect of pH ranging from 3.0 to 11.0 on the adsorption capacity (*q*_*e*_) of MA/RG–15% is shown in Fig. 6a. The amount of PNP adsorbed on MA/RG–15% increases with increasing initial pH of up to 5, and then decreases with further increase in pH. The effect of pH on PNP adsorption by MA/RG is mainly resulted from the distribution of two PNP species, the molecular and the anionic. The PNP is a water–soluble solid that is moderately acidic in water (*pK*_*a*_ = 7.15). The molecular PNP can be effectively adsorbed onto MA/RG at a lower pH by hydrophobic interaction and π–π interactions with the aromatic moieties of the PNP and the aromatic matrix of the MA/RG. However, when the pH value is below or equal to 4, a damage of MOFs starts to appear, leading to the weak binding force for PNP adsorption^42^. The PNP exists as a phenolate anion when the pH is higher than the *pK*_*a*_ (7.15) and is hydrophilic in nature, which cannot be effectively loaded onto MA/RG composite due to the weak electron accepting ability of benzene ring in PNP for donor–acceptor interaction with MA/RG. Furthermore, the zeta potentials measurement (Fig. 6a) shows that the surface charge of MA/RG is negative when pH is greater than or equal to 9, which implies that the electrostatic repulsion between PNP anions and the MA/RG surface increases with increasing pH value, resulting in the decrease of PNP uptake amount to MA/RG adsorbent.

Effect of presence of NaCl, KCl and BaCl_2_ on the PNP adsorption is depicted in Fig. 6b. It can be seen that the amount of PNP adsorbed on MA/RG is firstly decrease (or increase), and then increase (or decrease) with the increase in the concentration of NaCl, and BaCl_2_ (or KCl). This depends on the strength of two different interaction mechanisms. On the one hand, the Na^+^, K^+^ and Ba^2+^ are trended to compete with PNP for the active site of the MA via donor–acceptor interaction, making an adverse condition for PNP adsorption. On the other hand, a favorable factor is also provided by the salt–out effect between PNP molecule and salt. This effect illustrates that solubility of non electrolyte organic compounds in solution generally displays an inverse dependency on ionic strength. Namely, in the presence of dissolved ions, water is less ordered and compressible, and the cavity volume available to accommodate PNP molecules is reduced. Thus, the solubility of PNP neutral molecule is decreased in solution, and the more decrease in solubility, the more increase is in the sorption of PNP by enhanced diffusion^51^.

#### Recyclability of MA/RG and PNP adsorption mechanism

The regenerability of MA/RG-15% was examined using methanol and ethanol. As seen in Fig. 6c, the PNP adsorption performance of MA/RG-15% regenerated by methanol is superior to that renewed by ethanol. After five adsorption/desorption cycles using methanol, the PNP uptake amount still remains at 271.82 mg/g, indicating that the methanol is very suitable for the regeneration of MA/RG, and the MA/RG could be a cost-effective and promising adsorbents for PNP removal. To verify the adsorption mechanisms, the FT–IR spectra (Fig. 6d) of PNP, MA/RG, mixture of MA/RG and PNP solid, and the MA/RG loaded with PNP are conducted. For PNP, the triplet bands (689, 753 and 853 cm^–1^) are the ring C–H vibrations and the bands at 1101 and 1161 cm^–1^ are aromatic ring vibrations; the O–H bending vibration locates at the band of 1211 cm^–1^ (and 3080 ~ 3500 cm^–1^) and the phenolic C–O stretch is presented at 1337 cm^–1^; the bands at 1278 and 1442 cm^–1^ are due to the vibration of NO_2_ group; the bands at 1501 and 1594 cm^–1^ belong to the C–N band and aromatic stretching C=C vibrations, respectively^52^. As shown in Fig. 6d, the curve for the mixture of PNP and MA/RG (MA/RG + PNP) by mechanical blending is just a simple combination of the corresponding peaks of MA/RG and the PNP, no other obvious changes. Compare with the spectra of MA/RG + PNP, the MA/RG loaded with PNP (MA/RG adsorbed PNP) exhibits some distinctions, indicating that many interactions are established between the PNP and MA/RG during the adsorption process. As discussed in the spectra of RG, its surface also has the epoxy ether C–O–C at the edges of broken graphene planes, which could interact with the phenolic protons to form hydrogen bond. On the basis of the disappearance of the O–H stretch for PNP (Fig. 6d) after adsorption (MA/RG adsorbed PNP), as well as the position shift of C–O–C from 991 cm^–1^ for RG in MA/RG composite to 1000 cm^–1^ for MA/RG adsorbed with PNP, it is evident that there is hydrogen bonding between PNP and MA/RG composite^16^. The MIL–68(Al) contains numerous benzene rings with electron system, which possess the high affinity to attract the electron–withdrawing compounds by donor–acceptor interaction^40^. It is well known that such relationship can be affected by the availability of electron density in the donor and the electron affinity of the acceptor. The nitro–substituted benzene ring can acts as electron acceptor due to the low electron density caused by strong electron withdrawing ability of NO_2_ group reducing the overall electron density in the π–system of the aromatic ring^39^. As shown in Fig. 6e, the adsorption yield of nitrophenols and phenol onto MA/RG composite in case of single component is in the order of o–nitrophenol (ONP) > PNP > m–nitrophenol (MNP) ≫ phenol, indicating that the nitro substituent causes the increment in electronic acceptance of aromatic ring on nitrophenol. In fact, the π electron–rich regions in graphene layers also can interact with electron acceptor substance by π–π dispersion interaction^4^. For the multi aperture adsorbent, it is often assumed that the π−π interaction is stronger in the small pores. This agrees with the pore size distribution (Table 1) that the larger volume of micropore, the higher adsorption capacity is for PNP onto MA/RG samples. Besides, the C=C vibrations (Fig. 6d) in aromatic ring at 1587 and 1594 cm^–1^ for MA/RG and PNP are located in the uniform at the band of 1600 cm^–1^, indicating that the electron–rich regions in graphene layers interact with the π electron of the aromatic ring of PNP via stacking the center of the aromatic ring of the molecule on top of a graphene carbon atom and the benezene ring of the PNP on top of the graphene hexagon^18^. Owe to such force existence (Fig. 6f), it is possible to simultaneously remove isomeric nitrophenols from water. As presented in Fig. 6e, in the binary system (ONP + PNP, MNP + PNP), the MA/RG composite exhibits excellent affinity to each component with negligible change compared to that in single solution.

## Conclusions

The MIL–68(Al)/reduced graphene oxide (MA/RG) composite is successfully synthesized via a simple solvothermal method. The presence of reduced graphene oxide (RG) changes the morphology and surface area of composites, but not the crystalline structure. The surface area of composite is firstly increase and then decrease with increasing RG, and the PNP adsorption capacity exhibits the same trend with the maximum uptakes calculated from Langmuir model of 332.23 mg/g for MA/RG–15% at 303 K, which is much super than the MA and RG individual. This good performance is linked to improvement of porosity, the hydrogen bond and π–π dispersion interaction between PNP and the composite. The solution pH, ionic strength, temperature and initial PNP concentration extremely affect the PNP adsorption, but the presence of phenol and isomerism nitrophenols has a slightly influence on PNP removal. The adsorption process involved in film–diffusion and intra–particle diffusion beys well with the Pseudo–second–order model and the Langmuir model. The coupling of MOFs with reduced graphene oxide provides a favorable pathway to synthesis high reusability and effective adsorbent forsimultaneous removal of nitrophenols from wastewater.

## Methods

### Materials

Graphite powder (particle size <30 um) was purchased from Tianjin Kermel Chemical Regent Ltd (Tianjin, China). Terephthalic acid (99%), N, N–Dimethylformamide (DMF) and phenol (99.5%) were from Sinopharm Chemical Regent Co, Ltd (Shanghai, China). The AlCl_3_.6H_2_O was from Tianjin Hengxing Chemical Reagent Co, Ltd (Tianjin, China). The p–nitrophenol (PNP, 99.8%) was from Shanghai Shan Pu Chemical Co, Ltd (Shanghai, China). The o–nitrophenol (ONP, 98%) and m–nitrophenol (MNP, 99%) were from Xiya chemical industry Co. Ltd (Shandong, China). All other chemicals (analytical grade) were used without any other purification, and the ultrapure water resistivity at 18.3 MΩ.cm was used throughout the experiment.

### Synthesis of MIL–68 (Al)/reduced graphene oxide composites

The MIL–68(Al), or MA, was synthesized based on the method described by *Yang et al.*^23^. The graphene oxide was prepared by a modified Hummers method^53^. The MIL–68(Al)/reduced graphene oxide (MA/RG) composite was synthesized by a simple one-pot solvothermal method. Specifically, a certain amount of graphene oxide was added into 120 mL DMF and the mixture was sonicated for 30 min to form the brownish yellow suspension. Then 2 g of terephthalic acid and 1.95 g of AlCl_3_.6H_2_O were added. After the regents dissolved, the mixture was transferred to a 500 mL of single neck ground-in round flask with an Allihn condenser on top. The flask was set in an oil bath for reaction at 403 K 18.5 h. After that, the suspension was centrifuged, and the precipitation was washed with DMF and methanol four times, sequently. Finally, the product was dried at 423 K under vacuum for 3 h. The final products were labeled as MA/RG–1%, MA/RG–5%, MA/RG–15%, MA/RG–25% and MA/RG–35% with the ratio of RG to MIL–68(Al) at 0.01, 0.05, 0.15, 0.25 and 0.35, respectively. The reduced graphene oxide (RG) was synthesized via solvothermal method without terephthalic acid and AlCl_3_.6H_2_O addition.

### Characterization methods

The surface morphologies of the as–prepared materials were observed using the Environmental scanning electron microscope (SEM, FEI QuANTA 200, USA) equipped with an energy dispersive X–ray (EDX) spectroscopy, and Transmission electron microscopy (TEM, Tecnai G2F20 S–TWIN, USA). The X–ray diffraction (XRD) patterns were obtained using Bruker AXS D8 Advance diffractometer with Cu–Ka beam source (λ = 1.541 Å). Raman spectra were obtained using JobinYvon Micro–Raman Spectroscopy (RamLab–010), equipped with a holographic grating of 1800 lines/mm and a He–Ne laser (633 nm, spot size ∼ 1 μm) as excitation source. Fourier transform infrared spectrum (FT–IR) measurements were conducted by using Nicolet 5700 Spectrometer in KBr pellet at room temperature. The N_2_ adsorption–desorption for Brunauere–Emmette–Teller (BET) specific surface area and pore size measurement were conducted by using automatic surface analyzer (Quantachrome, USA). The surface elemental composition analyses were conducted based on the XPS spectra (Thermo Fisher Scientific–K–Alpha 1063, UK) with a resolution of 0.5 eV. The zeta potentials of MA/RG particles in solutions at pH from 3.0 to 11.0 (adjust by NaOH or HCl) was determined with a zeta potential meter (Zetasizer Nano–ZS90, Malvern).

### Adsorption of p**–**nitrophenol

Batch experiments were conducted to determine the kinetics and isotherms for p–nitrophenol (PNP) adsorption by MA/RG, and the factors affecting the adsorption as well. All the tests were performed in triplicates. For adsorption kinetics study, the 100 mg of adsorbents was added into 250 ml PNP solution with PNP concentration of 200 mg/L. The mixture was shaken on a gyratory shaker at 303 K and 160 rpm. At predetermined time intervals (from1 min to 24 h), 4 ml of mixture was withdrawn using a 5 ml of pipette. The experiments of adsorption isotherms were conducted at different temperatures (298, 308 and 318 K), for which 5 mg of adsorbents (RG, MA or MA/RG–15%) adding to 25 mL of solution with PNP concentration between 50 mg/L and 300 mg/L. The effect of pH on the PNP adsorption was examined by agitating the mixture of 5 mg of MA/RG–15% and 25 ml of PNP solution (100 mg/L) at 303 K under 160 rpm. The initial pH values of the PNP solutions were adjusted by using a pH meter (PHSJ–5, China) with 0.1 M NaOH or 0.1 M HCl. The test on influences of ionic strength was performed using 25 ml of 100 mg/L PNP solution containing 0 ~ 100 mmol/L of NaCl, KCl, or BaCl_2_ and 5 mg of MA/RG–15% at 303 K under 160 rpm. The influence of phenol, ONP or MNP on PNP adsorption at 303 K were investigated using 30 ml of the solution that contains 50 mg/L phenol (or ONP, or MNP) and 50 mg/L PNP and 10 mg of MA/RG–15%. For adsorption mechanism analysis, the solution containing only phenol, ONP, MNP, or PNP were used. The regeneration of MA/RG was conducted by agitating PNP-loaded-MA/RG in absolute methanol (or ethanol) with solid concentration of 500 mg/L at 303 K for 3h. Adsorption tests on renewed MA/RG were performed at 303 K in the solution containing 200 mg/L of PNP and 500 mg/L of MA/RG. After the solid–liquid separation at the end of adsorption, the concentrations of phenol, ONP, MNP and PNP in the supernatant was measured using UV spectrophotometer (UV–2550, SHIMADZU, Japan) at 270, 278, 273 and 317 nm, respectively. The adsorptive quantity was calculated as follows:


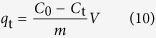


where *q*_*t*_ (*mg*/*g*) is the adsorption quantity; *C*_*0*_ and *C*_*t*_ are the pollution concentration of initial and interval time (*mg*/*L*), respectively; *V*(*L*) is the volume of solution, and *m* (*g*) is the weight of adsorbent.

## Additional Information

**How to cite this article**: Wu, Z. *et al.* Enhanced adsorptive removal of p-nitrophenol from water by aluminum metal-organic framework/reduced graphene oxide composite. *Sci. Rep.* **6**, 25638; doi: 10.1038/srep25638 (2016).

## Figures and Tables

**Table 1:** Parameters of the porous structure for the MA, MA/RG and RG.

Sample	S_BET_ (m^2^/g)	V_t_ (cm^3^/g)	V_mic_ (cm^3^/g)	V_mes_ (cm^3^/g)	V_mac_ (cm^3^/g)	Pore Size (nm)
RG	18.96	0.055	0.001	0.022	0.032	15.18
MA	550.03	0.220	0.002	0.096	0.122	21.38
MA/RG–1%	599.26	0.248	0.014	0.146	0.088	9.44
MA/RG–5%	629.52	0.259	0.013	0.144	0.102	9.26
MA/RG–15%	761.97	0.266	0.016	0.164	0.086	9.07
MA/RG–25%	714.77	0.231	0.015	0.118	0.098	9.24
MA/RG–35%	703.33	0.204	0.011	0.095	0.098	11.40

**Table 2:** Adsorption kinetics parameters of PNP onto adsorbents.

Kinetics	Parameters	Adsorbents						
		RG	MA	MA/RG −1%	MA/RG −5%	MA/RG −15%	MA/RG −25%	MA/RG −35%
Pseudo–first–order kinetic	*q*_*e,exp*_(*mg*/*g*)	137.63	187.13	203.50	243.38	307.38	247.13	234.75
	*k*_*1*_ (*1*/*h*)	0.19	0.14	0.18	0.09	1.79	0.21	0.10
	*q*_*e*_,_*cal*_ (*mg*/*g*)	67.92	77.10	113.27	134.91	152.10	108.97	99.62
	*R*^*2*^	0.865	0.925	0.979	0.965	0.964	0.907	0.799
Pseudo–second–order kinetic	*k*_*2*_(*g*/(*mg.h*))	2.04E–02	1.33E–02	8.77E–03	6.97E–03	7.85E–03	1.35E–02	1.59E–02
	*q*_*e,cal*_ (*mg*/*g*)	139.47	183.82	202.84	241.55	304.88	249.38	234.74
	*R*^*2*^	0.999	0.995	0.995	0.993	0.997	0.999	0.999
Elovich	*α* (*mg*/(*g.h*))	0.058	0.094	0.059	0.054	0.039	0.040	0.051
	*β* (*g*/*mg*)	3.47E + 03	7.08E + 06	6.26E + 04	3.74E + 04	1.23E + 05	3.19E + 04	1.70E + 05
	*R*^*2*^	0.988	0.942	0.924	0.883	0.981	0.992	0.996
Intra–particle diffusion	*k*_*1d*_ (*mg*/(*g.h*^*1*/*2*^))	112.89	78.96	51.12	47.21	150.90	115.37	93.72
	*C*_*1*_	11.44	94.04	82.86	110.04	108.00	76.87	97.01
	(*R*_*1*_)^2^	0.933	0.936	0.912	0.939	0.851	0.941	0.900
	*k*_*2d*_ (*mg*/(*g.h*^*1*/*2*^))	29.75	13.36	22.23	31.79	32.99	25.28	17.78
	*C*_*2*_	62.87	122.58	98.79	113.52	177.10	161.11	167.76
	(*R*_*2*_)^2^	0.985	0.973	0.989	0.991	0.965	0.983	0.969
	*k*_*3d*_ (*mg*/(*g.h*^*1*/*2*^))	0.19	9.94	5.60	11.51	10.07	0.278	2.51
	*C*_*3*_	136.75	139.05	176.42	188.02	258.61	245.76	222.52
	(*R*_*3*_)^2^	0.875	0.907	0.906	0.900	0.916	0.998	0.981

**Table 3:** Isotherm parameters for the adsorption of PNP onto adsorbents.

Adsorbents	T (*K*)	Langmuir				Freundich		
		*q*_*m.cal*_(*mg*/*g*)	*K*_*L*_(*L*/*mg*)	*R*_*L*_	*R*^*2*^	*1*/*n*	*K*_*F*_(*L*/*mg*)	*R*^*2*^
RG	303	175.44	0.04	0.072	0.998	0.18	58.55	0.997
	313	173.31	0.04	0.072	0.999	0.20	54.95	0.990
	323	175.75	0.03	0.10	0.996	0.25	40.65	0.986
MA	303	271.00	0.05	0.059	0.997	0.23	76.56	0.896
	313	305.81	0.05	0.069	0.993	0.27	69.46	0.908
	323	335.57	0.04	0.077	0.991	0.26	76.59	0.940
MA/RG	303	332.23	0.14	0.024	0.998	0.19	122.03	0.724
	313	336.70	0.16	0.021	0.998	0.19	129.51	0.681
	323	353.36	0.14	0.024	0.998	0.20	128.05	0.715

**Table 4:** Maximum adsorption capacities for PNP ontovarious adsorbents.

Sorbents	*T* (*K*)	*q*_*max*_ (*mg*/*g*)	Ref.
NiAl-layered double hydroxide	303	77.70	1
Alumina hollow microspheres	303	217.40	46
NH_2_-MIL-101(Al)	303	195.52	47
Copper-based MOFs (HKUST-1)	293	372.00	40
Carbon nanotube	293	206.00	3
Nanographite oxide	303	264.90	16
Graphene	298	15.50	18
Reduced graphene oxide	303	175.44	In this study
MIL-68(Al)	303	271.00	In this study
MIL-68(Al)/Reduced graphene oxide	303	332.23	In this study

**Table 5:** Thermodynamic parameters for PNP adsorption.

Adsorbents	*T*(K)	*Kc*	Δ*G*^*0*^ (kJ/mol)	Δ*S*^0^ (J/(K mol))	*ΔH*^*0*^ (kJ/mol)
RG	303	0.56	−3.38	−23.51	−10.57
	313	0.55	−3.34		
	323	0.48	−2.91		
MA	303	1.19	−4.90	53.41	11.36
	313	1.20	−5.21		
	323	1.26	−5.97		
MA/RG	303	1.93	−6.40	35.38	4.31
	313	2.05	−6.78		
	323	2.10	−7.11		
